# Are Downhill Varices an Overlooked Entity of Upper Gastrointestinal Bleedings?

**DOI:** 10.1155/2018/7638496

**Published:** 2018-07-31

**Authors:** M. A. Ayvaz, H. Rakici, H. D. Allescher

**Affiliations:** ^1^Department of Gastroenterology, Medical Faculty, Recep Tayyip Erdogan University, 53100 Rize, Turkey; ^2^Zentrum İnnere Medizin, Klinikum Garmisch-Partenkirchen, 82467 Garmisch-Partenkirchen, Germany

## Abstract

**Aim:**

Downhill varices are not so safe as thought and can lead to life-threating or mortal bleeding complication, even if rare. In order to draw attention to this topic, we analysed 129 patients.

**Materials and Methods:**

We evaluated the electronic endoscopy data records of all patients undergoing upper gastrointestinal endoscopy over a nine-year period from January 2004 till December 2012, within a retrospective approach. The primary endpoints, incidence, causes, kind of resulting upper gastrointestinal bleeding, and the severity of the bleeding were evaluated. Secondary endpoints were the evaluation of the size of downhill varices and a comparison of the risk of bleeding between downhill varices and uphill varices.

**Results:**

Downhill varices were identified, described, and/or documented in 129 patients of 25,680 upper gastrointestinal endoscopies. 26 patients had central venous catheter or port implantation, 22 patients had a history of an implantation of a cardiac pacemaker, 7 patients had severe pulmonary artery embolism, and 4 patients had severe chronic obstructive pulmonary disease. Two patients had mediastinal tumors, and one patient had a large retrosternal goiter as a possible cause of the vena cava syndrome. Altogether, 62 patients were related to a vena cava superior syndrome; 67 were not.

**Conclusions:**

Downhill varices can be seen with an incidence of 0.5%. Therapeutic means are the banding therapy as a safe and effective option. Severe bleedings associated with downhill varices can be mortal. Severe forms of downhill varices should be examined in relation to the origin in order to start a specific therapy.

## 1. Introduction

The first description of the esophageal varices located in the upper third of the esophagus without the existence of a portal hypertension was in 1964 by Felson and Lessure [1]. Since then, reports in the literature focusing downhill varices are mainly based on case reports and small series of cases. Downhill varices in the upper third of the esophagus occur as a consequence or increase of pressure in the vena cava superior or due to obstruction of the superior vena cava.

**Figure 1:**
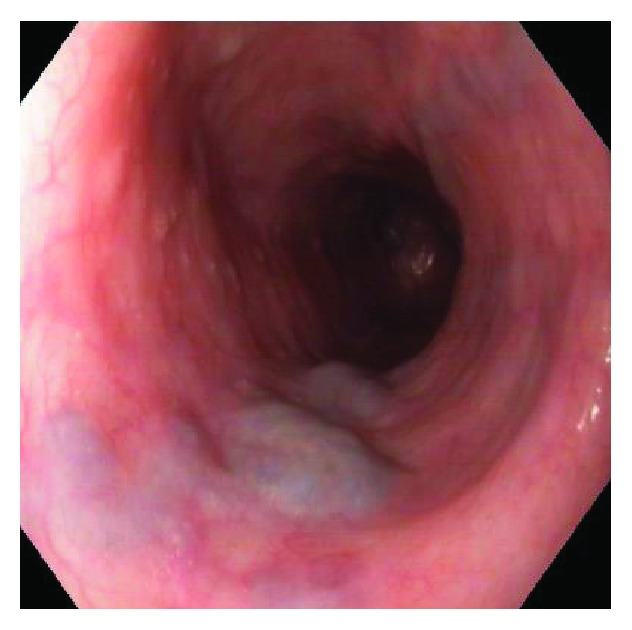
Type I downhill varices. Endoscopic appearance.

**Figure 2:**
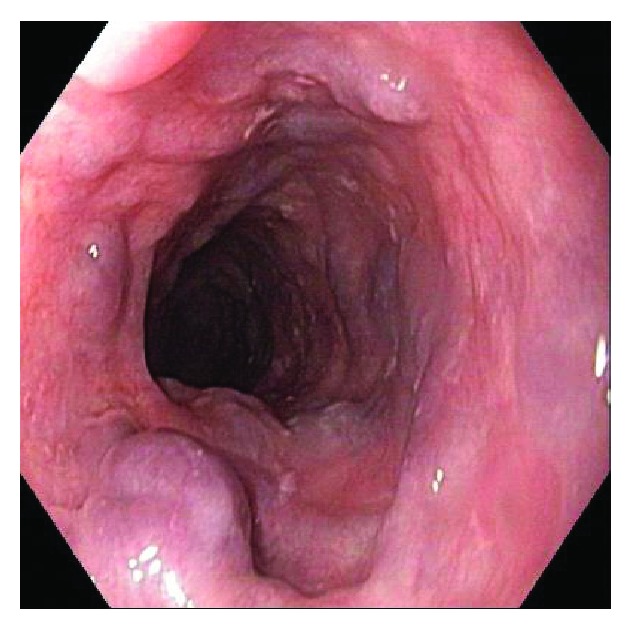
Type II downhill varices. Endoscopic appearance.

**Figure 3:**
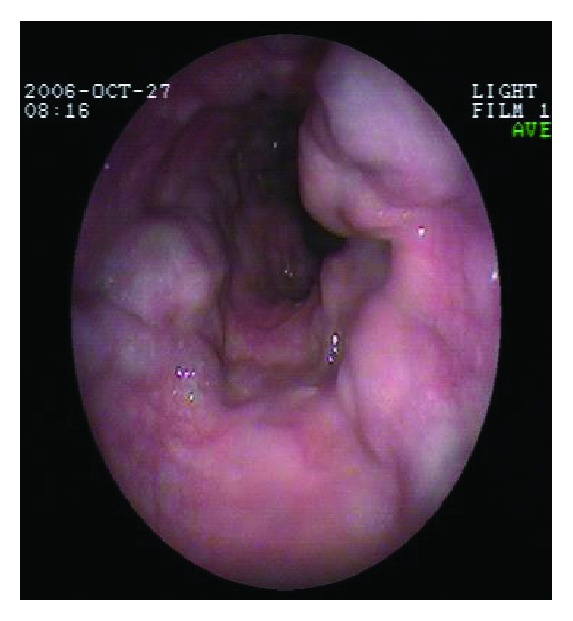
Type III downhill varices with an extent > 1/3 of the esophageal lumen. Endoscopic appearance.

In the following, the incidence and the clinical reasons of downhill varices are evaluated in a retrospective analysis. According to H. D. Allescher, endoscopically identified downhill varices were classified in three degrees (types I–III), whereas type I varices are defined as focal nodes above the mucosal level and type II varices are strands of veins over a distance of 2 cm but fill less than one-third of the esophageal lumen. Type III varices extend over more than 2 cm and fill more than one-third of the lumen (Table 1). Examples of this grading system are given in Figures 1–3.

There are only few systematic data on the origin, clinical significance, and especially therapeutic consequences of downhill varices. Causes of vena cava superior syndrome can also lead to downhill varices.

**Figure 4:**
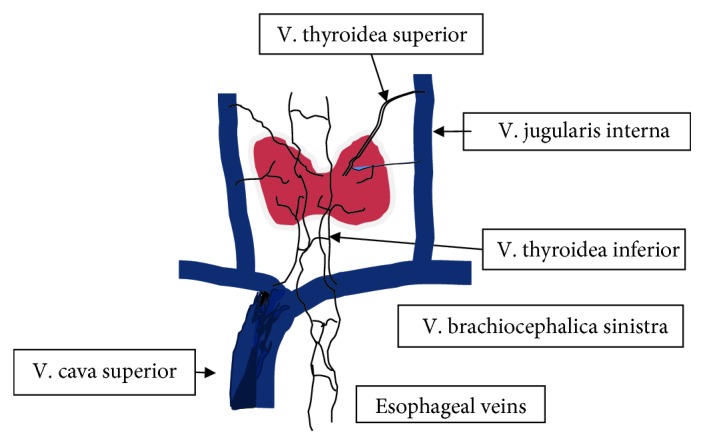
Schematic drawing of the collateral blood flow in the case of an occlusion of the vena cava superior. As shown, blood flows over the thyroid veins, then over the downhill varices of the esophagus and the azygos/hemiazygos vein into the inferior vena cava.

The venous drainage in the cervical part takes place over the V. thyroidea inferior (Figure 4). In the thoracic part, the drainage occurs over the V. azygos and hemiazygos, entering the V. cava superior above the entrance to the right atrium. The entrance to the right atrium is usually located on Th 4 from the incisors. Finally, in the abdominal part, the venous drainage occurs over the V. gastrica sinistra to the portal vein [2, 3].

Downhill varices develop as a consequence of a pressure increase or an obstruction of the vena cava superior in the upper third of the esophagus and are a rare cause of upper gastrointestinal bleeding. In this case, the blood flows from the V. cava superior over the V. azygos to the esophageal venous plexus, which is located in the mucosa and submucosa and then over the V. gastricae to the portal vein (Figure 4). Esophageal veins are normally not visible, except in cases, in which obstructive processes of the portal blood flow lead to a dilation of intramural and paraesophageal veins, which work as a collateral circulation between the portal vein and the azygos system and the vena cava system.

In contrast, “uphill” varices in the distal esophagus force the blood flow into the vena cava superior in the case of portal hypertension. The extent of downhill varices is dependent from the stage and the duration of the obstruction of the vena cava superior.

The weakness of the upper esophagial vein walls and motility disorders are also considered causes of downhill varices, but still, the etiology remains unknown. Causes of downhill varices are summarized in Table 2. A collection of the various reported causes of downhill varices are listed as case reports. In contrast to the esophageal varices associated with portocaval collaterals, bleeding complications are rare [4–7]. However, downhill varices should be examined further by magnetic resonance angiography/computer tomography of the thorax, because they often are associated with a severe diagnosis. Complications of downhill varices are mostly due to a stasis with edema, seldom to variceal bleeding and to pulmonary embolism. Therapeutic means are the banding therapy as a safe and effective option and sclerotherapy [8]. Other specific therapies targeting the cause of obstruction of the superior vena cava are thyroidectomy, chemoradiotherapy, and surgical resection [6–12].

The aim of this study was (a) to analyse the frequency, clinical causes, and significance of downhill varices detected during routine upper gastrointestinal endoscopy in an endoscopic center over a nine-year period, (b) to develop an endoscopic grading system of downhill varices, (c) to compare our finding with a review of all reported cases of downhill varices, and (d) to analyse these causes in connection with the underlying anatomy of esophageal vascular supply.

## 2. Patients and Methods

For our analysis, we evaluated in a retrospective approach the electronic endoscopy data records of all patients undergoing upper gastrointestinal endoscopy over a nine-year period from January 2004 till December 2012. All patients undergoing an upper gastrointestinal endoscopy because of other indications were documented with a standardized computer-based documentation system using the ESGE minimal standard terminology and video documentation. Since it is a retrospective study, informed consent form is not applied.

From 2004 to 2012, 25680 upper gastrointestinal endoscopies were performed and documented. According to internal standards, all abnormalities were documented with digital imaging and/or video, if necessary, in order to allow further evaluation. With a data-based search, all cases of downhill varices were identified and verified by the picture documentation and classified. The data analysis was approved by the Department of Study Review Board (IRB) and by the local ethics committee (Klinikum Garmisch-Partenkirchen).

All the upper gastrointestinal endoscopies were performed in the left lying position with the patient being sedated with 2 mg Midazolam (Dormicum; Roche) and 40–400 mg of Propofol (Propofol-Lipuro 1%; B. Braun Melsungen).

The procedures were performed with the following endoscopes: Olympus GIF-165 (2004–2006), GIF-H180 (2006–2012), and GIF-HQ 190 (2012), and documented digitally on the Clinic-Win-Data-System (E and L Computer Systems, Erlangen, Germany) with storing of about 8–20 pictures per procedure.

In all patients with downhill varices, the imaging data and the clinical history were used and evaluated, in order to identify the cause of the downhill varices. The primary endpoints, incidence, causes, kind of resulting upper gastrointestinal bleeding, and the severity of the bleeding were evaluated. Secondary endpoints were the evaluation of the size of downhill varices and a comparison of the risk of bleeding between downhill varices and uphill varices.

For the literature review, we identified all published manuscripts and case reports on downhill varices with the keyword downhill varices and upper esophageal varices. The publications were grouped, evaluated, and summarized. Statistical analyses were performed using power analysis and SPSS software, version 11.5.

## 3. Results

In the period from January 2004 to December 2012, 25,680 upper GI endoscopies were performed. Downhill varices were identified, described, and/or documented in 129 patients of 25,680 upper GI endoscopies. This corresponds to an incidence of 0.5%. After the review of the patients' digital clinical documents, 26 (20.1%) patients had interventional procedures (central venous catheter or port implantation), 22 (17%) patients showed a history of an implantation of a cardiac pacemaker, 7 (5.4%) patients had severe pulmonary artery embolism, and 4 (3.1%) patients had severe chronic obstructive pulmonary disease.

Further, 2 (1.6%) patients had mediastinal tumors and 1 (0.8%) patient had a large retrosternal goiter as a possible cause of a vena cava syndrome. Altogether, 62 patients showed a possible connection between the obstruction of the vena cava superior and consequent occurrence of downhill varices. The documents of the remaining 67 (52%) patients did not offer any information with a suspected relation. 43 (69.4%) of 62 patients with superior vena cava syndrome had first-degree downhill varices, 18 (29%) had second-degree varices, and 1 patient (1.6%) had third-degree varices. 48 patients (71.6%) of the 67 patients in the group without any relation to a superior vena cava syndrome had first-degree varices, 19 patients (28.4%) had second-degree varices. There were not documented cases of bleeding in both groups, neither there were differences regarding the size of the varices between the groups (*p* > 005). None of the patients had “uphill varices” in the distal esophagus nor cirrhosis of the liver.

We observed that there was not any significant increase in the size of the varices in 27 patients in the primary group, who were reendoscopied 5 years later. Bleeding was not detected in both groups. Our results are shown in Table 3.

## 4. Discussion

After the literature review, we found that there is not much research on this field regarding the etiology of the downhill varices. The greatest parts of the publications in literature are case reports. Most of the cases with superior vena cava syndrome are listed in the literature based on benign and malign origins.

Among the screened 25,680 patients, 129 (0.5%) patients had downhill varices. İn our study, 62 patients had superior vena cava syndrome, while 67 had not an obstruction of the superior vena cava. 98.4% of the patients with superior vena cava syndrome had benign underlying causes. The most frequent reasons reported in the literature are seen after implantation of cardiac pacemakers [13], venous catheters, dialysis catheters, port catheters, Behcets' disease, intrathoracic goiter [9], chronic obstructive pulmonary disease, pulmonary embolism, and positive lupus anticoagulants. A compression of mediastinal structures in the case of fibrosing mediastinitis can lead to the formation of downhill varices [7, 14]. Further, primary esophageal motor disorders should be considered a possible cause of ectatic veins in the proximal esophagus and “downhill” varices [10].

Rheumatoid and congenital heart diseases are listed as etiologic factors, too. Mediastinal tumors were described less; for example, bronchial-and thyroid carcinomas and large lymphomas in the mediastinum and metastases were possible reasons [8, 11, 12, 15–20].

In our study, the percentage of patients with underlying malign causes of superior vena cava syndrome associated downhill varices was low (1.6%). Regarding the size of the varices, there was not found any difference between the patients' groups with and without superior vena cava syndrome. There are publications relating to a downsizing of the varices after interventional procedures to a stenosis of the vena cava superior in patients with superior vena cava syndrome [4, 6–8]. The bleeding incidences showed up in literature are generally mild and exceptionally life threatening. According to a study, bleedings because of downhill varices represent 0.1% of all bleedings in the upper gastrointestinal tract [15]. The reported cases with bleeding had a superior vena cava syndrome. Additionally, some of the patients took anticoagulants or were under platelet antiaggregation therapy. Because of the fact that most of the patients with downhill varices and upper gastrointestinal bleeding were associated with superior vena cava syndrome, we can deduce that the existence of a superior vena cava syndrome is related more frequently with an upper gastrointestinal bleeding [9–18].

Screening the patients' documents in our retrospective study, there was no gastrointestinal bleeding in patients with downhill varices regardless of whether they had vena cava superior syndrome or not. A possible explanation could be that patients were endoscopied routinely because of other indications and downhill varices were found incidentally. If patients with superior vena cava syndrome were included in the study, in particular, the bleeding rate would have been higher. Because of lacking gastrointestinal bleeding among the screened cases, accordingly, there were not any therapeutical interventions done. The necessary interventions to solve the superior vena cava obstruction were performed by the concerned departments (interventional radiology, cardiology, vascular, and thorax surgery); for example, there was performed a stent implantation into the superior vena cava [4, 6]. In cases of primer downhill varices without upper gastrointestinal bleeding, treatment is not necessary. Additionally, as the primary cause is treated in patients with downhill varices related to vena cava superior syndrome, varices regress. Because it is out of concern, we do not refer. There were not any differences regarding the size of the varices between the groups. None of the patients had “uphill varices” in the distal esophagus nor cirrhosis of the liver. We observed that there was not any significant increase in the size of the varices in 27 patients in the primary group, who were reendoscopied 5 years later.

Downhill varices bleed less frequently than uphill varices. A possible explanation for this fact could be that portal hypertensive varices are related with coagulation disorders and are exposed to erosions because of gastroesophageal reflux. Furthermore, veins in the distal esophagus are running in the mucosal layer, whereas veins of the upper esophagus are submucosal.

The banding of the downhill varices is the therapeutic consequence. Although our long-term and wide-ranging study involves 25680 endoscopic scanning procedures, it is a retrospective study and there is no treatment applied by the authors. These factors can be seen as limitations.

The description of downhill varices is diagnostically useful and should be enforced, because almost 50% of the patients have a relevant underlying cause for downhill varices. In cases with none of the reported origins, a diagnostic workup with endoscopy/endosonography, duplex-sonography of the veins of the throat, transesophageal echocardiography, sonography of the thyroid/throat and computer-tomography of the thorax/throat (mediastinal tumors, venography), or nuclear magnetic resonance with reconstruction of vessels is recommended.

In conclusion, downhill varices can be observed in approximately 0.5% of all diagnostic routine upper gastrointestinal endoscopies. Prognosis is very good in primary downhill varices, but prognosis of downhill varices related to vena cava superior syndrome may be very poor. Treatment possibilities in the cases of bleeding of downhill varices are the band ligation and sclerotherapy.

Additionally, we can state that downhill varices conduct benign origins and rarely lead to clinical serious conditions. Nevertheless, it should memorized that it can rarely result in life-threatening bleeding complications.

## Figures and Tables

**Table 1:** Classification of the types of the downhill varices.

Type I varices: focal venous nodes above the mucosal niveau (Figure 1)
Type II varices: venous strand length > 2 cm, <1/3 of the esophageal lumen filled (Figure 2)
Type III varices: venous strand length > 2 cm, >1/3 of the esophageal lumen filled (Figure 3)

**Table 2:** Reported reasons for downhill varices (summary).

Superior vena cava syndrome:
(i) Thrombosis of the vena jugularis, truncus brachiocephalicus, and superior vena cava because of different reasons [16]
(ii) Fibrosing mediastinitis [13]
(iii) Venulitis, vasculo-Behcet's syndrome [7]
(iv) Dialysis catheter, atrial catheter [11]
(v) Central venous catheter [17]
(vi) Cardiac pacemaker implantation/cardiac synchronization therapy defibrillator [6]
(vii) Port implantation
(viii) Tumors of the upper mediastinum [8]:
(a) Retrosternal, intrathoracic goiter
(b) Thymoma
(c) Morbus Hodgkin
(d) Lymphoma [15]
(e) Carcinoma of the thyroid
(f) Bronchial carcinoma [16]
(g) Metastases (breast cancer, bronchialcarcinoma)
(i) Surgical ligature of the superior vena cava
(ii) Castleman syndrome [12]
(iii) Pulmonary hypertension
(iv) Hypercontractile esophagus motility disorders

**Table 3:** Etiological factors.

Central venous catheter or port implantation	26 (%20.1)
Cardiac pacemaker	22 (%17)
Chronic obstructive pulmonary disease	4 (%3.1)
Pulmonary artery embolism	7 (%5.4)
Mediastinal tumor	2 (%1.6)
Retrosternal goiter	1 (%0.8)
Not suspected relation	67 (%52)

## References

[1] Felson B., Lessure A. P. (1964). “Downhill” varices of the esophagus. *Diseases of the Chest*.

[2] Tamm E. R., Kurtz A. (2012). Anatomy and physiology of the esophagus. *Clinical Gastroenterology*.

[3] Floch M. H., Floch N. R., Kowdley K. V. (2009). Chapter: esophagus. *Netter’s Gastroenterology*.

[4] Gholam S., Ghazala S., Pokhrel B., Desai A. P. (2017). A rare case of downhill esophageal varices in the absence of superior vena cava obstruction. *The American Journal of Gastroenterology*.

[5] Nguyen L. P., Sriratanaviriyakul N., Sandrock C. (2016). A rare but reversible cause of hematemesis: “downhill” esophageal varices. *Case Reports in Critical Care*.

[6] Loudin M., Anderson S., Schlansky B. (2016). Bleeding ‘downhill’ esophageal varices associated with benign superior vena cava obstruction: case report and literature review. *BMC Gastroenterology*.

[7] Yasar B., Abut E. (2015). A case of mediastinal fibrosis due to radiotherapy and ‘downhill’ esophageal varices: a rare cause of upper gastrointestinal bleeding. *Clinical Journal of Gastroenterology*.

[8] Tavakkoli H., Asadi M., Haghighi M., Esmaeili A. (2006). Therapeutic approach to downhill-esophageal varices bleeding due to superior vena cava syndrome in Behcet’s disease: a case report. *BMC Gastroenterology*.

[9] Kelly T. R., Mayors D. J., Boutsicaris P. S. (1982). Downhill varices; a cause of upper gastrointestinal hemorrhage. *The American Surgeon*.

[10] Micklefield G. H., Schwegler U., Hüppe D., Wittenborg A., Wiebe V., May B. (1991). Circumscribed venous ectasia of the upper esophagus and downhill varices in primary disorders of esophageal motility. *Zeitschrift für Gastroenterologie*.

[11] Greenwell M. W., Basye S. L., Dhawan S. S., Parks F. D., Acchiardo S. R. (2007). Dialysis catheter-induced superior vena cava syndrome and downhill esophageal varices. *Clinical Nephrology*.

[12] Ichikawa M., Kobayashi H., Mukai M., Saitoh Y. (1991). Superior vena cava syndrome as initial symptom of Vasculo-Behçet’s disease-case report. *Nihon Kyōbu Shikkan Gakkai zasshi*.

[13] Basar N., Cagli K., Basar O. (2010). Upper-extremity deep vein thrombosis and downhill esophageal varices caused by long-term pacemaker implantation. *Texas Heart Institute Journal*.

[14] Basaranoglu M., Ozdemir S., Celik A. F., Senturk H., Akin P. (1999). A case of fibrosing mediastinitis with obstruction of superior vena cava and downhill esophageal varices: a rare cause of upper gastrointestinal hemorrhage. *Journal of Clinical Gastroenterology*.

[15] Areia M., Romãozinho J. M., Ferreira M., Amaro P., Freitas D. (2006). “Downhill” varices. A rare cause of esophageal hemorrhage. *Revista Española de Enfermedades Digestivas*.

[16] Hussein F. A., Mawla N., Befeler A. S., Martin K. J., Lentine K. L. (2008). Formation of downhill esophageal varices as a rare but serious complication of hemodialysis access: a case report and comprehensive literature review. *Clinical and Experimental Nephrology*.

[17] Serin E., Özer B., Gümürdülü Y., Yıldırım T., Barutçu O., Boyacioglu S. (2002). A case of Castleman’s disease with “downhill” varices in the absence of superior vena cava obstruction. *Endoscopy*.

[18] Shirakusa T., Iwasaki A., Okazaki M. (1988). Downhill esophageal varices caused by benign giant lymphoma: case report and review of downhill varices cases in Japan. *Scandinavian Journal of Thoracic and Cardiovascular Surgery*.

[19] Tanaka H., Nakahara K., Goto K. (1991). Two cases of downhill esophageal varices associated with superior vena cava syndrome due to lung cancer. *Nihon Kyōbu Shikkan Gakkai zasshi*.

[20] Hsu Y. H., Yang M. T., Hsia C. C., Tsai D. M. (2004). Esophageal varices as a rare complication of central venous dialysis tunneled cuffed catheter. *American Journal of Kidney Diseases*.

